# Supplementary material for: Abstract representations emerge naturally in neural networks trained to perform multiple tasks
Source: Nat Commun. 2023 Feb 23;14:1040. doi: 10.1038/s41467-023-36583-0 (PMC9950464; doi:10.1038/s41467-023-36583-0)
Supplement: Supplementary file 1 — Supplementary Information [file 41467_2023_36583_MOESM1_ESM.pdf]

Supplementary Methods

SM1 Four possibilities for representations in the multi-tasking model

We consider four distinct kinds of representations that could support the simultaneous performance of P classification tasks as formalized in the multi-tasking model. First, the high dimensional standard input could be preserved, or only weakly tuned – in particular, recall that high classification performance is already achieved on the standard input for random tasks (fig. 1f, left, standard) even though it is not abstract (fig. 1f, left, gen). Second, the representation could split along P separate dimensions of population activity, where each dimension corresponds to one of the P distinct tasks (fig. S1b, left). Third, the representation could consist only of an approximately D-dimensional sphere (or circle, in two dimensions), which exploits the correlation structure in the P different tasks (that is, when $P > D$, the outcomes from some pairs of tasks are necessarily correlated with each other; fig. S1b, middle). This second type of representation would have high classifier generalization performance but low regression generalization performance: That is, it is partially abstract in that it would recover the angular structure of the latent variables (as necessary for the P classification tasks), but not their magnitude (as this information is not necessary to solve the P tasks). Fourth, a fully abstract representation of the latent variables could be recovered. That is, the representation could recover both the angular structure of the latent variables, as in the second possibility, and their magnitude (fig. S1b, right). This would occur only if the multi-tasking model does not discard information about the stimuli that is not necessary for satisfying the tasks, but which is also not explicitly trained to discard. Surprisingly, as we will see, this fourth form of representation is most common in our trained networks, even for more disordered tasks than we have described so far.

SM2 Comparing the multi-tasking model with the unsupervised βVAE

We compare the level of abstraction of the representations learned by the multi-tasking model to those learned by an auto-encoder that is designed to produce abstract representations. In particular, the β-variational autoencoder (βVAE) is the current state-of-the-art for unsupervised disentangling of latent variables[1] (and it has many variations[2, 3]). It is designed around a hyperparameter, β, that is thought to control the trade-off between the abstractness of the representations in the latent variables and reconstruction error for output from the auto-encoder. That is, increasing β is understood to increase the level of abstraction in the βVAE representation layer, while decreasing the quality of of reconstruction of the original input representation.

Using the same architecture as in our multi-tasking model, we trained βVAEs to disentangle the same set of latent variables as in our other experiments. Applying the same two metrics as to our other models, we found that the βVAE produces moderately abstract representations, as quantified by the classifier generalization metric – though classifier generalization performance does not saturate to the same level as for the multi-tasking model. The βVAE does not produce high regression generalization performance for any choice of β that we tested. Because the multi-tasking model receives binary supervisory input and the βVAE does not receive any supervisory input at all, it is not particularly surprising that the multi-tasking model develops more abstract representations. However, we believe the contrast is still informative, as it indicates that abstract representations are unlikely to emerge by chance or without explicit training on tasks that are at least coarsely related to the latent variables of interest (and see [4]). Further, this multi-tasking approach to producing abstract representations is less sensitive to changes in model and input parameters than the βVAE

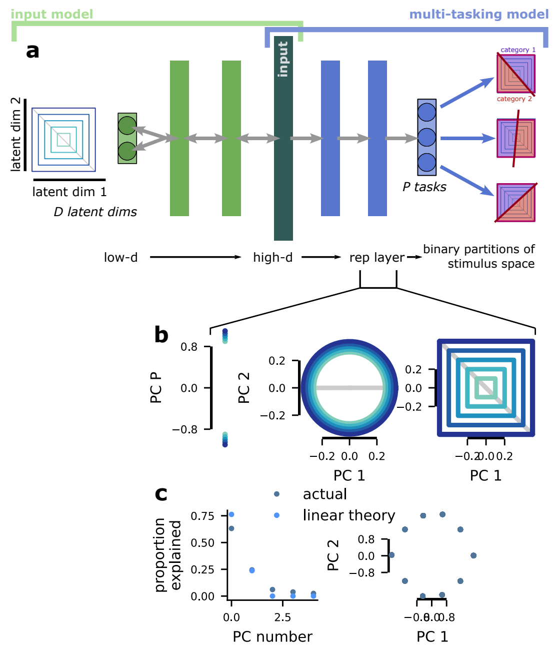

Figure S1: Possibilities for learned representations. **a** Schematic of the multi-tasking model. It receives an entangled stimulus representation (left) and learns to perform P binary classifications of the latent variables (right). We study the representations that this induces in the layer prior to the output: the representation layer. **b** Different possible solutions the network could learn. (left) The network could learn a dimension for each classification task and develop binary representations along each of those P dimensions. (middle) The network could learn a surface that matches the dimensionality D of the latent variables, but discards information about magnitude; this representation would have high classifier- but low regression-generalization performance. (right) The network could learn a fully abstract, approximately D-dimensional representation of the latent variables. **c** The dimensionality of the representation layer will be approximately D-dimensional (left), as predicted by eq. (3). (right) The first two principal components of the required output of the network; this structure is consistent with both the middle and right network solutions from **b**.

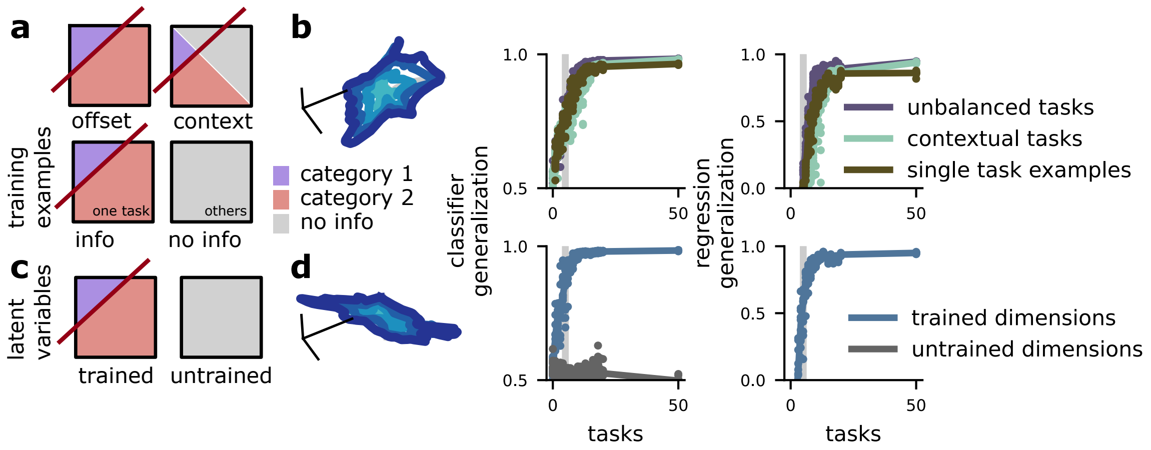

Figure S2: Abstract representations emerge for heterogeneous tasks, and in spite of high-dimensional grid tasks. **a** Schematics of different task manipulations. **b** (left) Visualization of the representations developed for contextual tasks $P = 25$. (middle) Classifier generalization performance. (right) Regression generalization performance. **c** Schematic showing the training scheme: A subset of latent variables are involved in tasks (left), the rest of the latent variables are not (right). **d** (left) Visualization of the trained latent variable representations. (middle) Classifier generalization performance for the trained and untrained latent variable dimensions. (right) Regression generalization performance for the trained and untrained latent variable dimensions.

(see A sensitivity analysis of the multi-tasking model and βVAE in). This further indicates the feasibility of the multi-tasking approach in conditions similar to those found in the brain.

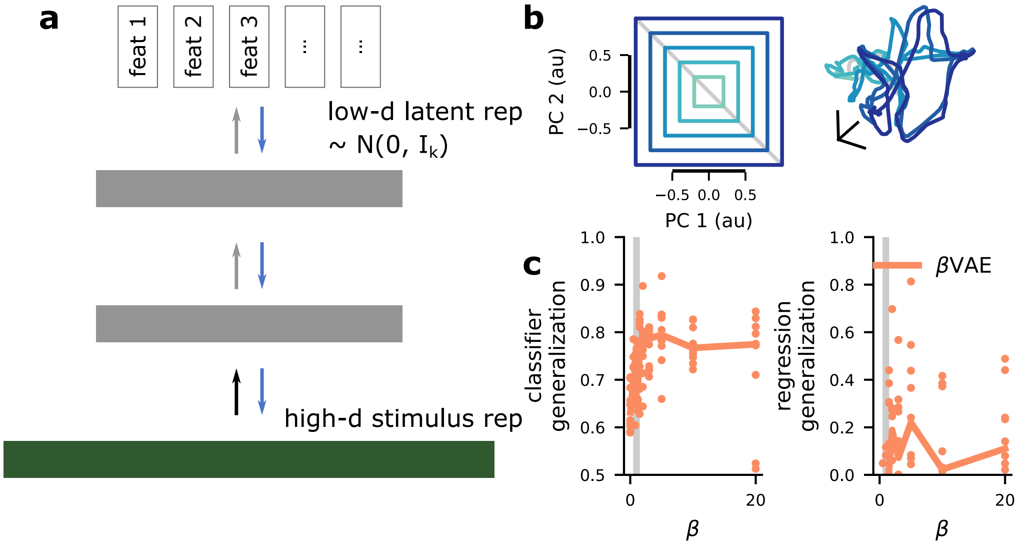

Figure S3: The βVAE does not reliably produce abstract representations. **a** A schematic of the βVAE. It is an autoencoder regularized to produce a low-dimensional representation in its representation layer. It receives the same high-dimensional standard input as the multi-tasking model in fig. 3. **b** A purely auto-encoding approach with the βVAE is not supplied with any classification tasks (left), but does produce moderately abstract representations (right). **c** The βVAE produces high classifier generalization performance for a small range of βs (left), but does not provide high regression generalization performance for any choice of β that we tested (right).

SM3 Abstract structure can be learned from early sensory-like representations.

While we explored highly nonlinear representations in the main text using low length scale random Gaussian process inputs, we also explored a special case: Gaussian receptive field inputs, which may be present in early sensory and other areas.

Here, we construct a representation of a $D = 5$ latent variable using Gaussian receptive fields (fig. S4a) which induces a curved geometry in population space (fig. S4b). While this format is lower dimensional than the dimensionality-maximized input used previously, it is constructed to have no global structure (i.e., each neuron responds only to a local region of latent variable space). We visualize the consequences of this structure as for the standard input (fig. S4c). Then, quantify the level of abstraction present in the input represent using our two metrics. First, almost any binary classification of the input space can be implemented with high accuracy, but the classifier generalization performance is near chance (fig. S4d, left). This is a consequence of the lack of global structure in the representation. The regression metric follows this same pattern: While the standard performance of a linear regression is relatively high, the regression generalization performance is at chance (fig. S4d, right).

Now, we train the multi-tasking model with these receptive field-like representations as input. First, we visualize the representations produced by the multi-tasking model as before (fig. S4e,f). Then, we quantify the level of abstraction for different numbers of trained tasks (fig. S4g). The metrics show that the representation becomes fully abstract, with above-chance classification and regression generalization performance. However, the regression generalization performance never

becomes close to its maximum value.

Representation details

Here, we use uniformly distributed latent variables to simplify the choice of an appropriate width for the RF population. For each unit in the input, we randomly choose the location of its center in the full D-dimensional latent variable space. Then, the unit's response is given by,

$$\mathrm{RF}_i(\mathrm{x}) = \exp\left(-\sum_{j}^{D} \frac{(x_j - \mu_{ij})^2}{w^2}\right)$$

where x is a D-dimensional stimulus, μ_i is the RF center for unit i, and w is the width of the RF. We choose the width to both achieve full coverage of the latent variable space and maximize the Fisher information of the code, following the procedure described in [5].

SM4 The multi-tasking model can be used as an abstract, generative model

In the main text, we show that the multi-tasking model produces abstract representations from two image datasets used in machine learning. One of the main applications of abstract (or, "disentangled") representations in machine learning is the generation of novel images with particular latent variable values – as from the βVAE[6].

Here, we demonstrate that the multi-tasking model can also be used in this generative context, to produce images with expected latent variable values from the 2D shape dataset. In addition, we compare the performance of the multi-tasking model to the βVAE. Importantly, the multi-tasking model is supplied with categorical information that is related to the latent variables, as it is throughout the paper, so this comparison does not put the βVAE and multi-tasking model on equal footing; the βVAE is designed to develop abstract representations in a fully unsupervised setting. Further, we also modify the multi-tasking model, as described before, to add an autoencoder. Now, the multi-tasking model is trained to both satisfy the P classification tasks as well as reconstruct the original image sample to test its generative properties.

We selected one shape to be left out as a test shape, and then used the representations corresponding to the other two shapes to learn a linear regression that decodes shape scale. We then used this linear regression to generate images of one of the trained shapes at different scales (fig. S5e,f, top row). Both models retained a reasonable degree of shape structure as well as produced an increase in scale, moving from left to right in the images shown. Next, we attempted to apply the learned representation of scale to the left out shape. Again, both models produce shapes with an increase in scale (fig. S5e,f, bottom row). However, while the multi-tasking model produces images with the left out shape, the βVAE does not represent a differentiated shape at all. This issue with the βVAE has been reported before: To achieve a high level of abstraction in the representations, the βVAE often sacrifices precision in its reconstruction of the target image[1] (but see [2]). In contrast, the multi-tasking model produces abstract representations while still preserving its ability to reconstruct different shapes from this dataset.

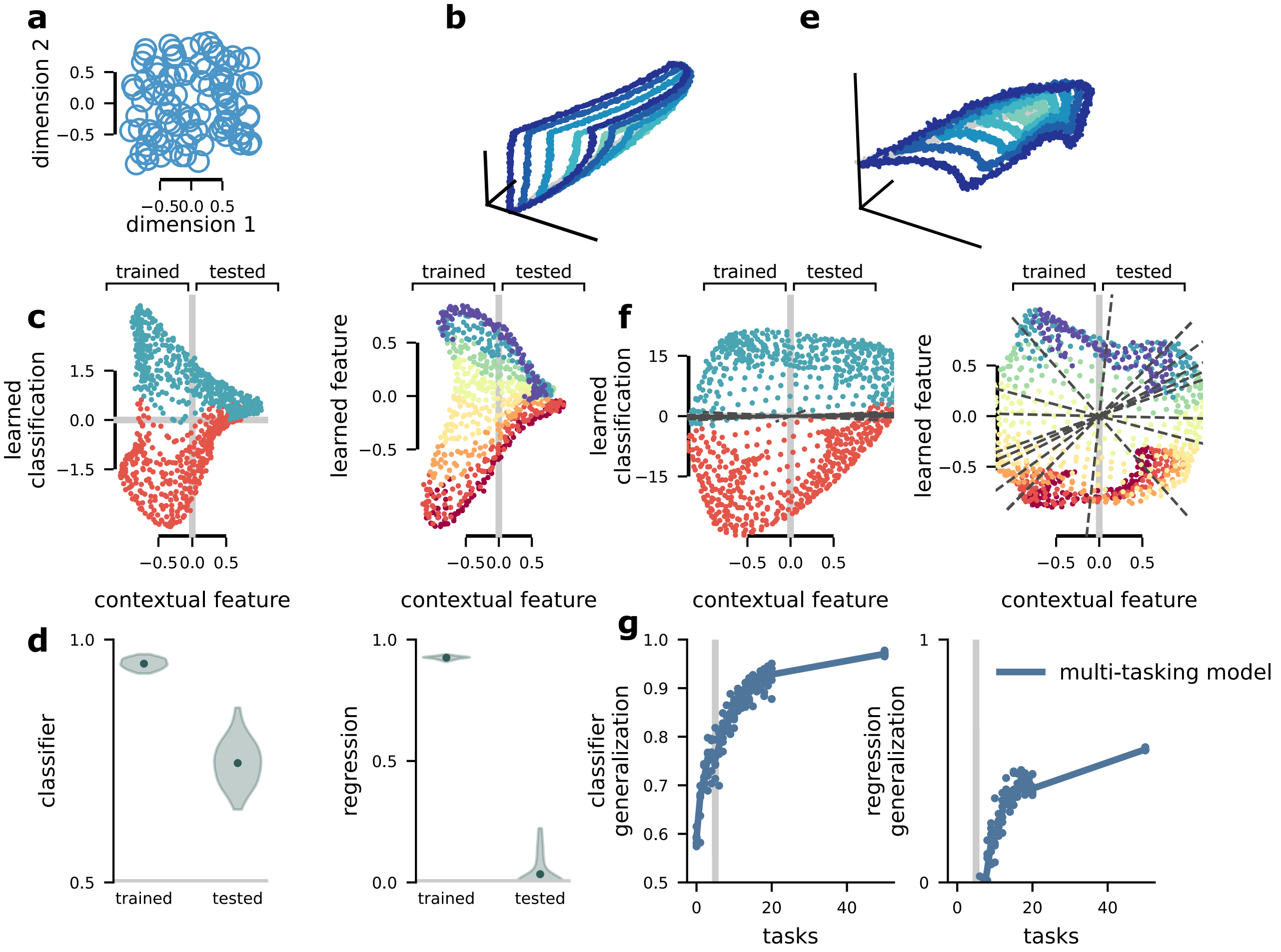

Figure S4: Abstract representations can be recovered even from highly nonlinear stimulus representations. **a** Schematic of the receptive field inputs. They are randomly arranged with the width chosen to both achieve coverage of the full latent variable space and minimize errors. **b** Visualization of the RF input representation, as done for the standard input in fig. 2c. **c** Visualization of the ability of a classifier (left) and regression (right) to generalize across a changing contextual feature (x-axis), same analysis as fig. 2e. **d** Quantification of **c**. **e** Visualization of the representation learned by the multi-tasking model on the RF inputs, as in fig. 3b. **f** Visualization of the ability of a classifier (left) and regression (right) to generalize across a changing contextual feature (x-axis) using the representation of an multi-tasking model trained on $P = 10$ tasks, same analysis as fig. 3c. **g** Quantification of **f**, same analysis as fig. 3e.

The parameters of the generative multi-tasking model

To move our multi-tasking model into a generative context, we simply add a series of layers connected to the representation layer that are trained to reproduce the original stimulus. Our objective function then has two parts: The first is to satisfy the training classification tasks and the second is to reconstruct the original input, as with a traditional autoencoder. The generative multi-tasking model was trained on the 2D shapes dataset (which were resized to be 32×32 images) with the following parameters,

layer widths	128x2x2, 128x2x2, 512, 256, 128, 128
representation width	50
batch size	30
training examples	100000
epochs	200

For the reconstruction part of the model, the given layer list is reversed.

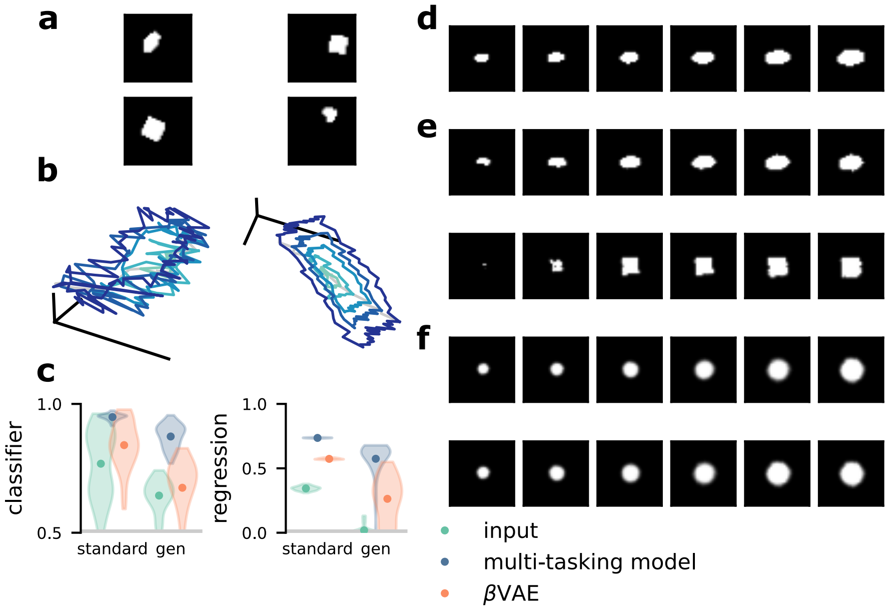

Figure S5: The multi-tasking model can be used for compositional image generation. **a** Example images from the 2D shapes dataset. **b** Visualization of the image representation manifold for x- and y-position from the representation layers of the multi-tasking model (left) and the βVAE (right). **c** Quantification of the abstractness of the original dataset (left points), the multi-tasking model (middle points, $P = 50$), and the βVAE (right points, $\beta = 1$) according to both our classifier- (left) and regression-generalization (right) metrics. In each plot, performance when training and testing on the whole stimulus set is on the left, and training and testing on separate halves is on the right; chance for both is shown by the grey line. **d** An example traversal of the scale dimension from the image set. **e** Image reconstruction for the multi-tasking model with a shape that was present in the training set (top) and that was held out from the training set (bottom). **f** The same as **e** but for the βVAE. Notice that the βVAE loses the shape information in both rows, though it does preserve the scaling.

SM5 The dependence of learned abstract representations on latent variable dimensionality

For both the multi-tasking model and βVAE, our simulations reveal that abstract representations are more readily and consistently produced for more latent variables (fig. S6). Initially, we believed that this was due to a higher ratio of dimensionality expansion (i.e., the ratio between the participation ratio of the resulting input representation and the number of latent variables). However, we manipulated this while holding the number of latent variables constant and found similar levels of abstraction for higher and lower ratios (fig. S10).

Instead, we believe that there are at least two other features at work: The multiple tasks learned by the multi-tasking model during training specify the location of each sample on the surface of a D-dimensional sphere. As the number of latent variables increases, random samples from their distribution will also tend to be located closer and closer to the surface on a D-dimensional sphere. This agreement could increase abstraction. Second, we observe that, for a given novel task, one way in which the multi-tasking model can successfully generalize is by already having learned a task that is correlated with the novel task and uncorrelated with the contextual split. In lower latent variable dimensions, the previously learned tasks are likely to all be significantly correlated with both the novel task and the contextual split. In higher, dimensions, it becomes more likely that the learned tasks are correlated with one of the novel task or the split and not both. This could

also contribute to better abstraction performance for larger numbers of latent variables. However, developing a full understanding of this phenomenon is beyond the scope of the current work.

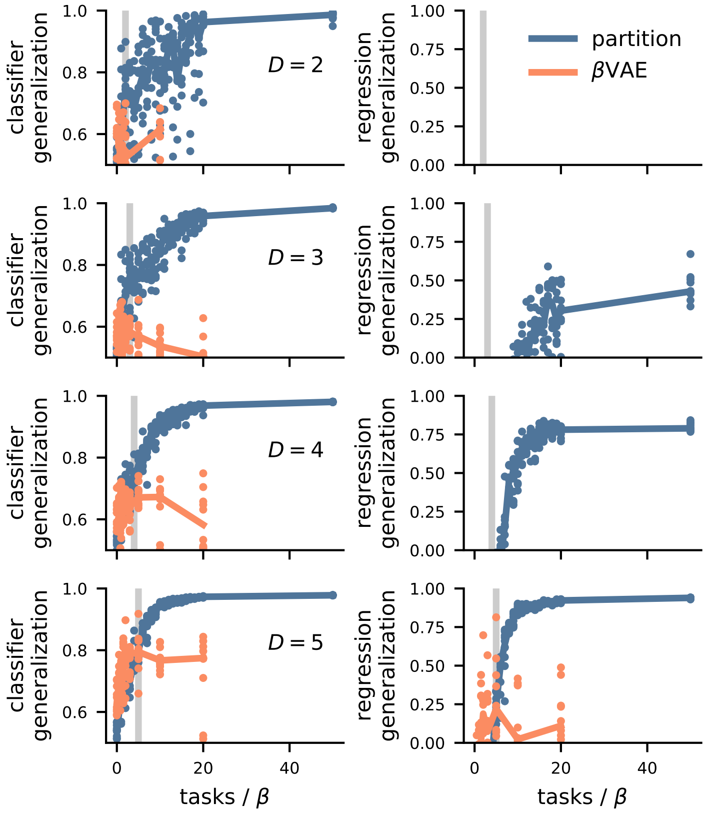

Figure S6: Abstraction learning depends on latent variable dimensionality. (top to bottom) Increasing latent variable dimensionality D, from $D = 2$ to $D = 5$ (see left inset). (left) Classifier generalization performance as a function of the number of classification tasks for the multi-tasking model and β for the βVAE. (right) Regression generalization performance as a function of the number of classification tasks for the multi-tasking model and β for the βVAE.

SM6 A sensitivity analysis of the multi-tasking model and βVAE

While we have focused on manipulation of the number (and kind) of classification tasks provided to the multi-tasking model and to the value of β for the βVAE, both models depend on numerous other parameter choices, which were essentially arbitrary. The parameters were held constant across the two models, but these choices can still affect the results produced by both models in different ways. To explore the dependence of our results on these other parameter choices, we performed a multiverse sensitivity analysis[7]. That is, for many of the parameters of our models, we chose several other similarly reasonable parameter values, and trained models with those parameters (e.g., using a tanh nonlinearity instead of the ReLU). In exploring this parameter space, we defined 7128 and 3369 distinct combinations of parameters for the multi-tasking model and βVAE respectively. Then, for each of these parameter combinations, we trained two models of the corresponding type and averaged their classification and regression generalization performance. The parameters varied for each were the same except for the choices of the number of classification tasks, values of β, and we included a version of the multi-tasking model with and without an autoencoder. To analyze these results, we fit linear models with ridge regression to account for the classification and regression

generalization performance from the different parameter choices. Using only the first order version of this model (that is, without fitting interaction terms for the different parameters), the model has $r^2 = .62$ and $r^2 = .70$ for the multi-tasking model and βVAE, respectively. As expected, for the multi-tasking model, the number of classification tasks has by far the strongest effect on both classification and regression generalization performance (fig. S7a,b), though minor effects on both are produced by almost all the other parameter choices – and the regression generalization metric is strongly affected by the dimensionality of the latent variables (fig. S7b). Surprisingly, while choice of β does affect classification and regression generalization performance for the βVAE, the size of the effect is similar in size to the effects associated with many of the other parameters – and much smaller than the increase in both classification and regression generalization performance that is produced by using a tanh nonlinearity rather than a ReLU nonlinearity.

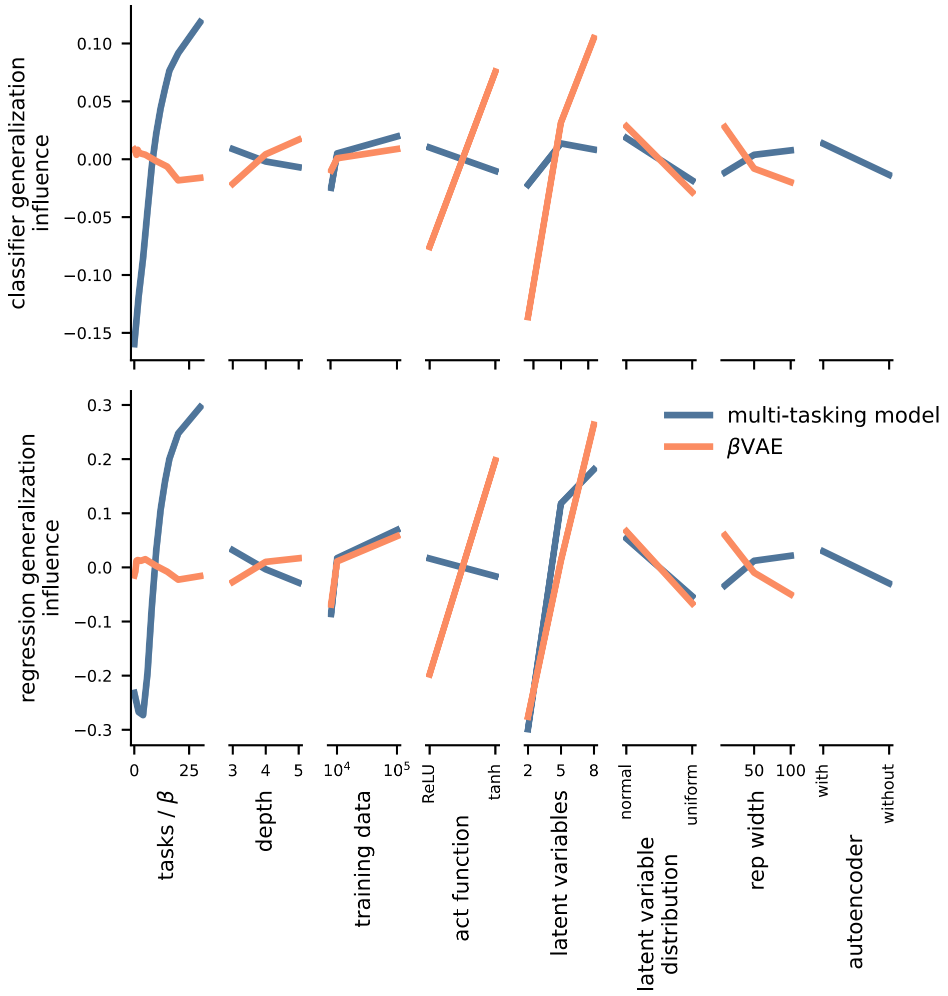

Figure S7: A multiverse analysis of both the multi-tasking model and βVAE. (top) The effects of different parameter choices on classifier generalization performance. (bottom) The effects of different parameter choices on regression generalization performance.

SM7 Zero-shot categorical generalization for image inputs

For the image inputs used in the main text, we also exploit the fact that they are described by a mixture of continuous and categorical features to explore other tests of generalization. In particular, while both sets of images were described by three close-to-continuous features (both: x- and

y-position, chairs: 3D rotation, shapes: size), they both also had a fourth categorical feature: chair and shape type (see fig. S8a,c for examples). First, we trained the multi-tasking model using only a subset of the shapes (or chairs) and then characterized the classification and regression generalization performance using a completely novel set of shapes (chairs, fig. S8a). The shapes have high classification and regression generalization performance and the chairs have high classifier generalization performance but low regression generalization performance for this test of generalization (fig. S8b).

Then, we test another form of zero-shot generalization. As before, we train the multi-tasking model on only a subset of shapes (chairs). Then, when evaluating the classification and regression generalization performance, we train the models on that same set of shapes (chairs) in a restricted section of the latent variable space (as usual), and then evaluate the performance on those models on both the held out shapes (chairs) and the left out section of the latent variable space (fig. S8c). In this case, the shape dataset has high classification and regression generalization performance while the chair dataset has high classifier generalization performance but chance-level regression generalization performance (fig. S8d). Thus, even for this strong test of generalization to unseen images, the multi-tasking model succeeds at producing fully abstract representations of the shape dataset and partially abstract representations of the chair dataset.

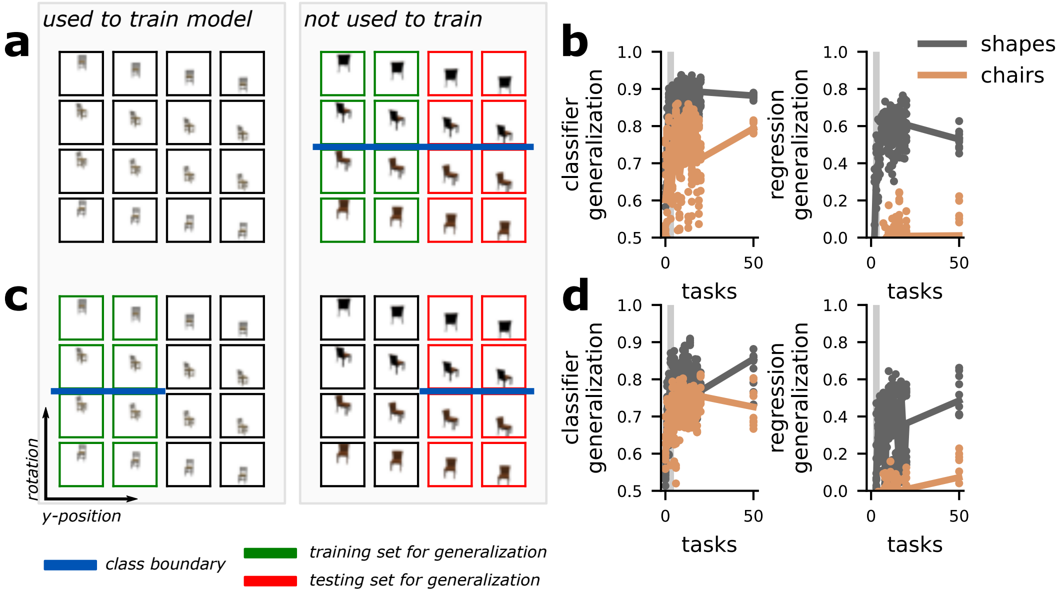

Figure S8: Tests of zero-shot generalization to novel images. **a** Schematic of the model training (boxes) and generalization analysis (red, green highlights) procedures for the first test of generalization. **b** Generalization performance for the training procedure shown in **a**. **c** Same as **a** for a second type of generalization analysis. **d** Generalization performance for the training procedure shown in **c**. We have used the shape[8] and chair[9] datasets from the main text here as well. The 2D shapes dataset is from: Matthey, L., Higgins, I., Hassabis, D. & Lerchner, A. dsprites: Disentanglement testing sprites dataset. https://github.com/deepmind/dsprites-dataset/ (2017).

SM8 The effect of activity regularization on abstraction

To further understand the robustness of our findings, we trained the multi-tasking model with various levels of both L1 and L2 regularization applied to the activations in the representation layer. Further, both of these procedures are likely to encourage the representations to be sparse (as defined in Quantifying sparseness and dimensionality in Methods)– and therefore, at least in this key way, more similar to the kinds of neural representations that we see in the brain.

We find that both forms of regularization dramatically increase the sparseness of activity in the representation layer (fig. S9a, c). However, both kinds of regularization also moderately decrease the abstraction of the resulting representations (fig. S9b, d), as measured by our classification and regression generalization metrics. This is expected: More sparse representations will tend to be higher dimensional, and therefore will tend to have lower abstraction. However, this finding also shows that for moderate levels of regularization, the representations developed by the multi-tasking model are both relatively sparse and relatively abstract.

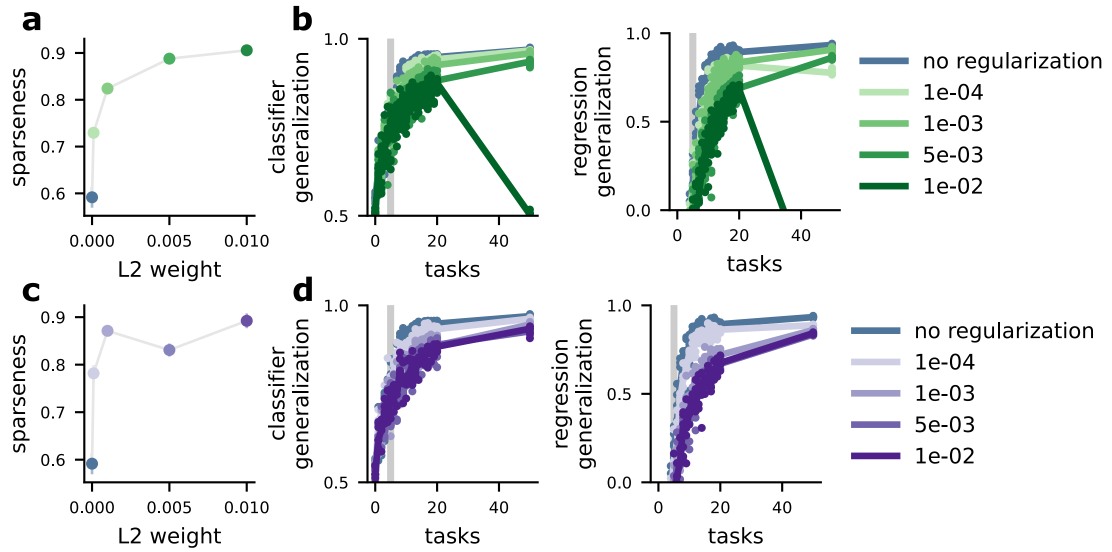

Figure S9: Activity regularization increases representation sparseness and moderately decreases abstraction. **a** Sparseness in the representation layer of multi-tasking models trained with different levels of L1 activity regularization. **b** (left) Classifier generalization performance as a function of the number of classification tasks for the multi-tasking models trained with different levels of L1 regularization (levels are same as in **a**). (right) Regression generalization performance as a function of the number of classification tasks. For higher regularization values and large numbers of tasks, the representation collapses and there is no activity in the representation layer – which causes chance-level generalization performance. **c** Same as **a** but for L2 regularization. **d** Same as **b** but for L2 regularization.

SM9 The effect of increased input dimensionality on abstraction

We investigated whether increasing the dimensionality of the input would reduce the ability of the multi-tasking model to learn abstract representations. To manipulate the dimensionality of the input, we trained the standard input as previously described but altered the number of units in its output layer. As we increased the number of units, both the dimensionality (participation ratio, fig. S10a) and the sparseness of the representations (fig. S10b) increase. However, this increased dimensionality and sparseness causes only a small decrease in the classification and regression generalization performance of multi-tasking models trained on these higher dimensional inputs (fig. S10c).

SM10 The effect of constant layer widths on abstraction

We asked whether or not including a decrease in layer width relative to the input (i.e., a structural bottleneck that would force lower dimensional representations) in the layers of the multi-tasking model was essential to its abstract representations. We trained a multi-tasking model with two hidden layers, both of width 500 as well as a representation layer of width 500, the same as the standard

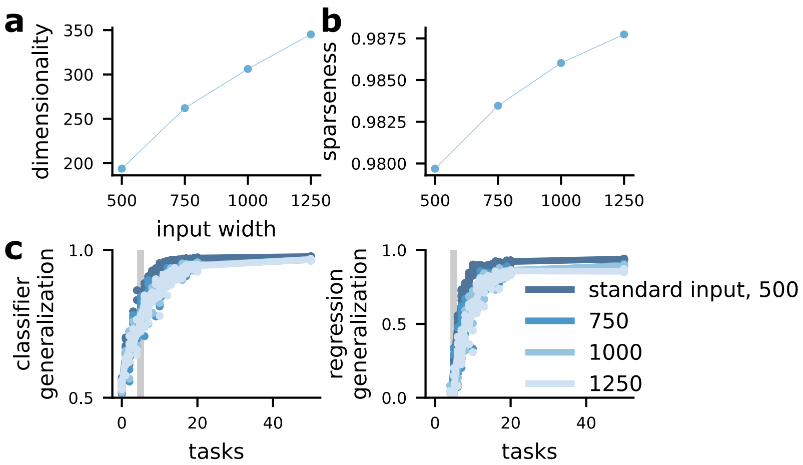

Figure S10: Increased input width has only a moderate effect on the abstraction developed by the multi-tasking model. **a** Dimensionality of the standard input as a function of input dimensions. **b** Sparseness of the standard input as a function of input dimensions. **c** (left) Classifier generalization performance as a function of the number of classification tasks for multi-tasking models trained on standard input with different widths. (right) Regression generalization performance as a function of the number of classification tasks.

input. We found that this model develops representations with the same level of abstraction as the multi-tasking model with the layer structure used elsewhere (fig. S11).

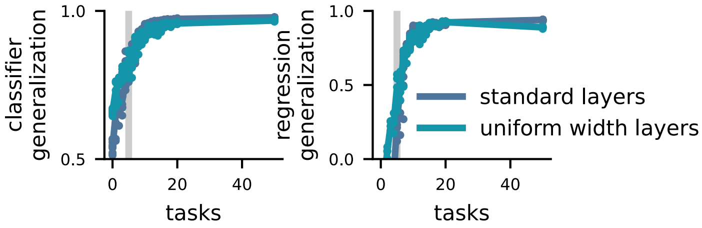

Figure S11: A multi-tasking model with uniform layer widths has the same level of abstraction as the nominal model. (left) Classifier generalization performance as a function of the number of classification tasks for multi-tasking models trained with either the standard layer widths used elsewhere in the paper or constant widths for all layers, set to be the same width as the input (500). (right) Regression generalization performance as a function of the number of classification tasks for the same models.

SM11 Abstraction emerges even in earlier layers of the multi-tasking model

We have explored several different numbers of hidden layers for the multi-tasking model and found high levels of abstraction for all of them. So, we asked whether abstract representations also develop in earlier layers of the multi-tasking model or only close to the output, in the representation layer we have studied so far. We find that in both multi-tasking models with the standard width and with uniform widths, abstract representations develop after the first hidden layer, after only one nonlinear step (fig. S12a, b). However, the classifier generalization performance slightly increases in the second hidden layer (fig. S12a, b). The representation layer has the same classification and regression generalization performance as the prior hidden layer, which is expected because it is a linear transform of the second hidden layer.

This result is somewhat surprising, but follows the intuition: Representations with high embedding dimensionality (such as the standard input) can be directly transformed to abstract representations when supplied with appropriate training information (here, the outcomes of several classification tasks).

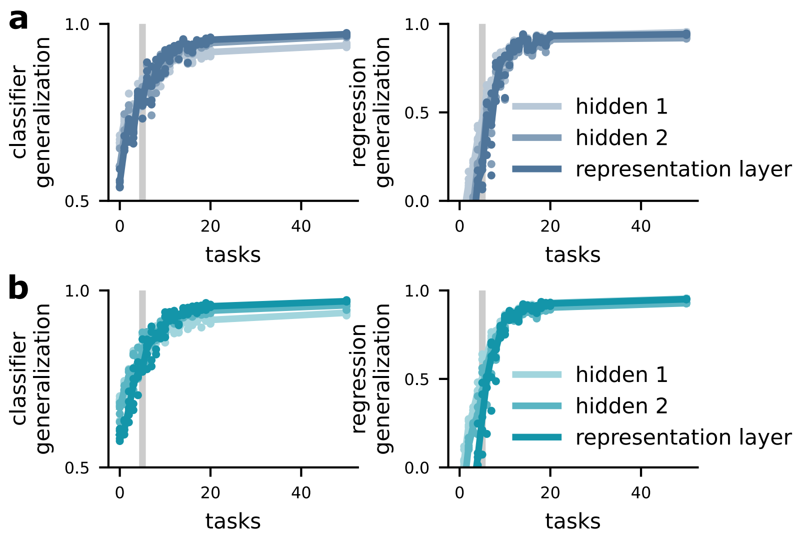

Figure S12: Abstraction emerges even in earlier layers of the multi-tasking model. **a** (left) Classifier generalization performance as a function of the number of classification tasks for different layers of the multi-tasking model, with nominal layer widths. (right) Regression generalization performance as a function of the number of classification tasks. **b** Same as **a** except the multi-tasking model has uniform layer widths of 500, matching the width of the input.

SM12 Novel random Gaussian process task learning

We reproduce the analysis shown in fig. 3f using a novel random Gaussian process task rather than a novel linear task, as used before. In this case, we also use a support vector machine decoder with a nonlinear, radial basis function kernel. Thus, the decoder should be capable of reaching close to perfect performance on the novel task, despite its nonlinear category boundary. Again, we begin by establishing an upper bound on performance (fig. S13, light grey), produced by training the decoder with the given number of samples directly on the latent variables, as well as a lower bound (fig. S13, dark grey), that is derived from training the decoder directly on the standard input. Across three different length scales, we find that the multi-tasking model representation can be learned to use the novel task significantly better than from the input.

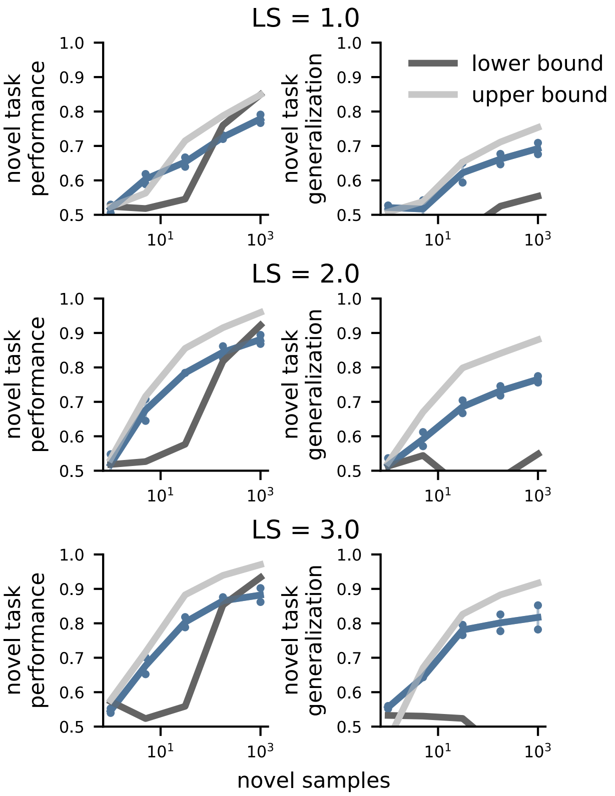

Figure S13: The learning of a novel random Gaussian process task is both sample efficient and generalizable. The task performance and generalization performance for a novel task learned with few samples (as in fig. 3f), except the novel task is generated using a random Gaussian process. The results are shown for several different length scales and compared to an upper bound, the performance of a decoder learning the novel task from the latent variables, and a lower bound, the performance of a decoder learning the novel task from the input, as before (fig. 3f). The multi-tasking model is trained to learn $P = 10$ linear tasks. The multi-tasking model comes close to saturating the upper bound in many cases.

Supplementary References

1. Higgins, I. *et al.* *β-VAE: Learning basic visual concepts with a constrained variational framework* in *ICLR* (2017).
2. Burgess, C. P. *et al.* Understanding disentangling in β-VAE. *arXiv preprint arXiv:1804.03599* (2018).
3. Kim, H. & Mnih, A. *Disentangling by factorising* in *International Conference on Machine Learning* (2018), 2649–2658.
4. Locatello, F. *et al.* *Challenging common assumptions in the unsupervised learning of disentangled representations* in *international conference on machine learning* (2019), 4114–4124.
5. Johnston, W. J. & J, F. D. Redundant representations are required to disambiguate simultaneously presented complex stimuli. *bioRxiv,* 2022–12 (2022).
6. Higgins, I. *et al.* beta-vae: Learning basic visual concepts with a constrained variational framework. *arXiv* (2016).
7. Steegen, S., Tuerlinckx, F., Gelman, A. & Vanpaemel, W. Increasing transparency through a multiverse analysis. *Perspectives on Psychological Science* **11,** 702–712 (2016).
8. Matthey, L., Higgins, I., Hassabis, D. & Lerchner, A. *dSprites: Disentanglement testing Sprites dataset* https://github.com/deepmind/dsprites-dataset/. 2017.
9. Aubry, M., Maturana, D., Efros, A. A., Russell, B. C. & Sivic, J. *Seeing 3d chairs: exemplar part-based 2d-3d alignment using a large dataset of cad models* in *Proceedings of the IEEE conference on computer vision and pattern recognition* (2014), 3762–3769.
