# Supplementary material for: Abstract representations emerge naturally in neural networks trained to perform multiple tasks
Source: Nat Commun. 2023 Feb 23;14:1040. doi: 10.1038/s41467-023-36583-0 (PMC9950464; doi:10.1038/s41467-023-36583-0)
Supplement: Supplementary file 3 — Reporting Summary [file 41467_2023_36583_MOESM3_ESM.pdf]

nature portfolio

Corresponding author(s): Johnston WJ; Fusi S

Last updated by author(s): Dec 21, 2022

Reporting Summary

Nature Portfolio wishes to improve the reproducibility of the work that we publish. This form provides structure for consistency and transparency in reporting. For further information on Nature Portfolio policies, see our Editorial Policies and the Editorial Policy Checklist.

Statistics

For all statistical analyses, confirm that the following items are present in the figure legend, table legend, main text, or Methods section.

n/a	Confirmed	
☐	☒	The exact sample size (n) for each experimental group/condition, given as a discrete number and unit of measurement
☐	☒	A statement on whether measurements were taken from distinct samples or whether the same sample was measured repeatedly
☒	☐	The statistical test(s) used AND whether they are one- or two-sided<br>*Only common tests should be described solely by name; describe more complex techniques in the Methods section.*
☐	☒	A description of all covariates tested
☒	☐	A description of any assumptions or corrections, such as tests of normality and adjustment for multiple comparisons
☐	☒	A full description of the statistical parameters including central tendency (e.g. means) or other basic estimates (e.g. regression coefficient) AND variation (e.g. standard deviation) or associated estimates of uncertainty (e.g. confidence intervals)
☒	☐	For null hypothesis testing, the test statistic (e.g. F, t, r) with confidence intervals, effect sizes, degrees of freedom and P value noted<br>*Give P values as exact values whenever suitable.*
☒	☐	For Bayesian analysis, information on the choice of priors and Markov chain Monte Carlo settings
☒	☐	For hierarchical and complex designs, identification of the appropriate level for tests and full reporting of outcomes
☒	☐	Estimates of effect sizes (e.g. Cohen's d, Pearson's r), indicating how they were calculated

Our web collection on statistics for biologists contains articles on many of the points above.

Software and code

Policy information about availability of computer code

Data collection	The data in this manuscript all comes from simulations done using open source software. The software used includes: python (3.x), tensorflow (2.x), numpy (1.22.x), scipy (1.8.x), scikit-learn (1.x), and pandas (1.3.x).<br><br>The code is available at the following link: https://github.com/wj2/disentangled
Data analysis	All of the data analysis was done using the same open source software.<br><br>The code is available in the following github repository: https://github.com/wj2/disentangled

For manuscripts utilizing custom algorithms or software that are central to the research but not yet described in published literature, software must be made available to editors and reviewers. We strongly encourage code deposition in a community repository (e.g. GitHub). See the Nature Portfolio guidelines for submitting code & software for further information.

Data

Policy information about availability of data

All manuscripts must include a data availability statement. This statement should provide the following information, where applicable:

- Accession codes, unique identifiers, or web links for publicly available datasets
- A description of any restrictions on data availability
- For clinical datasets or third party data, please ensure that the statement adheres to our policy

The large-scale simulation data generated in this study have been deposited in the Figshare database and at the following link: https://doi.org/10.6084/m9.figshare.21761348.v1

More detail about how to use these data to generate the figures is provided in the following github repository: https://github.com/wj2/disentangled

Human research participants

Policy information about studies involving human research participants and Sex and Gender in Research.

Reporting on sex and gender	n/a
Population characteristics	n/a
Recruitment	n/a
Ethics oversight	n/a

Note that full information on the approval of the study protocol must also be provided in the manuscript.

Field-specific reporting

Please select the one below that is the best fit for your research. If you are not sure, read the appropriate sections before making your selection.

☒ Life sciences ☐ Behavioural & social sciences ☐ Ecological, evolutionary & environmental sciences

For a reference copy of the document with all sections, see nature.com/documents/nr-reporting-summary-flat.pdf

Life sciences study design

All studies must disclose on these points even when the disclosure is negative.

Sample size	Since all experiments were computational, we had full control over sample sizes. In most cases, we found that the N = 10 intializations of the same class of model was sufficient to show the heterogeneity in results that arises from different initializations.
Data exclusions	We did not exclude any model results from the groups of N = 10 simulations above.
Replication	We found our results to be very consistent across different initial conditions. We also performed an extensive parameter sweep analysis to test whether the results also depend on other parameters. The full results of this analysis are shown in the supplement. In brief, while there were minor effects from other hyperparameters, the main explanatory variable we focus on in this study (the number of tasks the model is trained to perform) still correlates with the development of abstract representations for these other hyperparameter choices.
Randomization	There were no experimental groups since these were computational experiments.
Blinding	There was no blinding. We did not feel it was necessary since we were using an established analysis method to analyze the representations developed in computational models, and our extensive hyperparameter search demonstrates that our results are robust to many alternative choices that we could have made in model design.

Reporting for specific materials, systems and methods

We require information from authors about some types of materials, experimental systems and methods used in many studies. Here, indicate whether each material, system or method listed is relevant to your study. If you are not sure if a list item applies to your research, read the appropriate section before selecting a response.

Materials & experimental systems

n/a	Involved in the study	
☒	☐	Antibodies
☒	☐	Eukaryotic cell lines
☒	☐	Palaeontology and archaeology
☒	☐	Animals and other organisms
☒	☐	Clinical data
☒	☐	Dual use research of concern

Methods

n/a	Involved in the study	
☒	☐	ChIP-seq
☒	☐	Flow cytometry
☒	☐	MRI-based neuroimaging
